# Bare surface of gold nanoparticle induces inflammation through unfolding of plasma fibrinogen

**DOI:** 10.1038/s41598-018-30915-7

**Published:** 2018-08-22

**Authors:** Bahar Kharazian, Samuel E. Lohse, Forough Ghasemi, Mohamad Raoufi, Amir Ata Saei, Fatemeh Hashemi, Fakhrossadat Farvadi, Reza Alimohamadi, Seyed Amir Jalali, Mohammad A. Shokrgozar, Nasser L. Hadipour, Mohammad Reza Ejtehadi, Morteza Mahmoudi

**Affiliations:** 1 Department of Chemistry, Tarbiat Modares University, P. O. Box, 14115-175 Tehran, Iran; 2 Physical and Environment Sciences Program, Colorado Mesa University, Grand Junction, Colorado, 81501 United States; 3 Department of Chemistry, Sharif University of Technology, Tehran, 11155-9516 Iran; 4 Department of Nanotechnology and Nanotechnology Research Center, Faculty of Pharmacy, Tehran University of Medical Sciences, Tehran, Iran; 5 Department of New Materials and Biosystems, Max Planck Institute for Intelligent Systems, Heisenbergstraße 3, D-70569 Stuttgart, Germany; 6 Division of Physiological Chemistry I, Department of Medical Biochemistry and Biophysics, Karolinska Institutet, Scheelesväg 2, SE-17 177 Stockholm, Sweden; 7 Department of Immunology, School of Medicine, Shahid Beheshti University of Medical Sciences, Tehran, Iran; 8 Departman of Immunology, Shahid Beheshti University of Medical Sciences, Tehran, Iran; 9 National Cell Bank, Pasteur Institute of Iran, Tehran, Iran; 10 Department of Physics, Sharif University of Technology, P. O. Box 11155-9161, Tehran, Iran and Center of Excellence in Complex Systems and Condensed Matter (CSCM), Sharif University of Technology, Tehran, 1458889694 Iran; 11 Department of Anesthesiology, Brigham and Women’s Hospital, Harvard Medical School, Boston, Massachusetts, 02115 United States

## Abstract

The surface of nanoparticles (NPs) get coated by a wide range of biomolecules, upon exposure to biological fluids. It is now being increasingly accepted that NPs with particular physiochemical properties have a capacity to induce conformational changes to proteins and therefore influence their biological fates, we hypothesized that the gold NP’s metal surface may also be involved in the observed Fg unfolding and inflammatory response. To mechanistically test this hypothesis, we probed the interaction of Fg with gold surfaces using molecular dynamic simulation (MD) and revealed that the gold surface has a capacity to induce Fg conformational changes in favor of inflammation response. As the integrity of coatings at the surface of ultra-small gold NPs are not thorough, we also hypothesized that the ultra-small gold NPs have a capacity to induce unfolding of Fg regardless of the composition and surface charge of their coatings. Using different surface coatings at the surface of ultra-small gold NPs, we validated this hypothesis. Our findings suggest that gold NPs may cause unforeseen inflammatory effects, as their surface coatings may be degraded by physiological activity.

## Introduction

Upon entry to any biological environment, a nanoparticle’s (NP’s) surface is spontaneously masked with a layer of proteins and other biomolecules, leading to the formation of the so-called “protein corona”^1^. As protein corona can affect NP targeting^2^ and exert immune-active conformational changes in the interacting proteins^3^, probing the interaction of individual proteins with the surface of NPs and their corresponding biological identity are one of the central challenges in the field of nanomedicine and have attracted a great deal of attention^4^.

Fibrinogen (Fg) is one of the most abundant blood proteins, and plays a crucial role in immune activation and blood clotting processes. With a diameter of 5 nm and a length of 45 nm^5^, Fg consists of six polypeptide chains, (α; β; γ)_2_, which are held by disulfide-bridges^6^. Each chain consists of two outer D domains, which are connected to the central E domain by a coiled-coil segment^6^. Lishko *et al*. indicated that Fg has a binding site (γ^377–395^) in the D-domain for the Mac-1 receptor (CD11b/CD18 or α_M_β_2_) and that conformational changes adjust the α_M_β_2_-binding site in Fg and the subsequent inflammatory response^7^. Because of Fg’s ubiquity in serum and the potential inflammatory response due to Fg mis-folding, Fg-nanoparticle interactions are an important area of study in nanotherapeutics. Minchin *et al*. revealed that negatively charged PAA(poly (acrylic acid))-coated gold NPs have a capacity to induce the unfolding of Fg and expose sequences of amino acids in the C-terminus of γ chain, resulting in inflammatory cytokine release^3^. Their study suggested that the PAA coating was the main player in induction of the inflammatory response. It should be noted, however, that the surface coatings of NPs are complicated chemical systems, and other physiochemical factors could influence the binding of Fg at the NP surface.

The protein-NP interactions leading to corona formation are not limited to electrostatic attractions; under certain conditions, proteins may penetrate the organic molecules on the NP surface and interact with the NP core. For example, it is known that the PAA brush structure can be remarkably swollen by increasing temperatures (from 20 °C to 40 °C), creating open space for proteins (e.g., serum albumin) to homogeneously penetrate to the NP coating layer^8^. Therefore, the main hypothesis of this study is that the observed unfolding of the Fg protein by 5 nm negatively charged PAA-coated gold NPs could be due to penetration of Fg in the PAA brushes and its accessibility to the surface of gold NPs. In other words, we hypothesized that both the bare surface of gold NP’s and specific organic coatings are responsible for the observed inflammatory response.

To test this hypothesis, we used molecular dynamics (MD) simulations, which is a precise approach to understand the interaction of bio-nano materials at the molecular level^3^. The empirical force field used in MD simulations has been already employed to describe the time evolution of bond lengths, bond angles and torsions, also the non-bonding van der Waals and electrostatic interactions between atoms for a number of chemical systems, including NPs^9–12^. For instance, Agashe *et al*.^13^ studied the conformational changes of Fg over a self-assembled monolayer using AMBER force field, and reported that surface chemistry has the main role in controlling the orientation and conformation of the adsorbed proteins to the surface of NP’s.

It is increasingly being accepted that the integrity of different types of molecular coatings is strongly dependent on the size of NP’s^14–16^. For the ultra-small NP’s (e.g., ~10 nm or smaller), the significant curvature of the particle surface may open up gaps in the molecular coating layer. The presence of these gaps in the coating layer increases the possibility of Fg interaction with the bare surface of gold NPs. Therefore, one may expect that regardless of the nature and surface charge of the NP coating, some of the Fg proteins may have access to the bare surface of gold NP’s. This access may be due either to gaps in the surface coating or the dissolution of the coating in biological media, which may induce protein mis-folding and hence inflammatory responses.

In this study, we used MD to investigate the interaction of Fg protein with PAA coated- and bare-gold surfaces. The results revealed that, besides PAA coating, the bare gold surface also has a significant role in inducing inflammatory-responsive Fg conformational changes. More specifically, we found that the bare surface of Au(111) is the dominating factor in exposing the C-terminal amino acid sequences (γ^377–395^) of Fg protein. To complement the MD study, we then experimentally probed the interaction of Fg with gold NP’s possessing two different surface coatings (2-hydroxypropane-1,2,3-tricarboxylate (citrate) and cetyltrimethylammonium bromide (CTAB)), which provide different surface charges and coverage on the ~5 nm gold NP’s. The Fg-gold NP interactions were studied using circular dichroism and absorbance spectroscopy. The outcomes of this study demonstrated that both CTAB and citrate gold NPs could induce changes in Fg secondary structure, leading to inflammatory responses, and supporting the MD findings on the critical role of bare gold surfaces in Fg-NP interactions. As a very recent report revealed that the coating of gold NPs may be removed by enzymatic activities *in vivo*^17^, this finding may increase concerns on the long-term inflammatory and/or toxicity effects of gold NPs.

## Results

The main objective of this study was to mechanistically explore the interaction of coated gold NPs with plasma Fg, and to show which of the structural/compositional aspects of coated gold NPs, is the most influential in governing their interactions with plasma Fg and more importantly, changing its conformation. Although, it has been already reported that the coatings on the surface of gold NPs might be responsible for such a phenomenon, there is a body of evidence that these coatings are partially degraded *in vitro* and *in vivo*^17^. Ding *et al*.^18^ developed a simple model using discrete molecular dynamics with Medusa^19^ force field to investigated interaction between AgNP and ubiquitin to obtain general properties of the molecular system. They only modeled the surface atoms using a coarse-grained interaction between the core and the protein. Since the physical properties of this simulation are general, they just have captured the effect of small spherical nanoparticles on ubiquitin conformations^18^. But biomolecules induce polarization on gold atoms in adsorption process^20^ which has not been considered in their model^18^. Stefano Corni *et al*.^20^ using computational simulation developed GOLP-CHARMM force field to identify the dynamic polarization of gold atoms and the interaction between sp^2^ hybridized carbon atoms and gold. According to Ling *et al*.^21^, Au(111) is mostly exposed plan in gold NP conjugarets^21^. Also Siming Zhang showed the most stable structure for AuNPs larger than 2 nm is truncated-octahedral structure (fcc)^22^. Therefore, we hypothesize γ chain of Fg that its active site is located in the γ chain of Fg^3^ and its interaction with bare gold NP surface can be partially responsible for conformational changes induced in Fg. We performed all atoms MD simulation using GOLP-CHARMM force field^20^ to support this hypothesis. As a matter of fact, MD simulations pointed to the bare NP surface as the most influential parameter in gold NP-induced conformational changes in plasma Fg. However, as the integrity of the capping agents’ multi-layers of monolayers on the surface of ultra–small NP’s can be easily breached (due to pre-existing gaps in the coating layer, capping agent dissolution, or changes in PAA conformation), we also hypothesized that the detected unfolding of Fg by the bare gold surfaces might also be partially induced by other coating molecules, regardless of their composition and surface charge. As such, we designed empirical experiments based on gold NP’s with different ligand coatings (i.e. with smaller, more labile capping agents possessing different surface charges and coverages compared to PAA) to investigate this phenomenon in more detail.

### Molecular Dynamic Simulation

**Figure 1:**
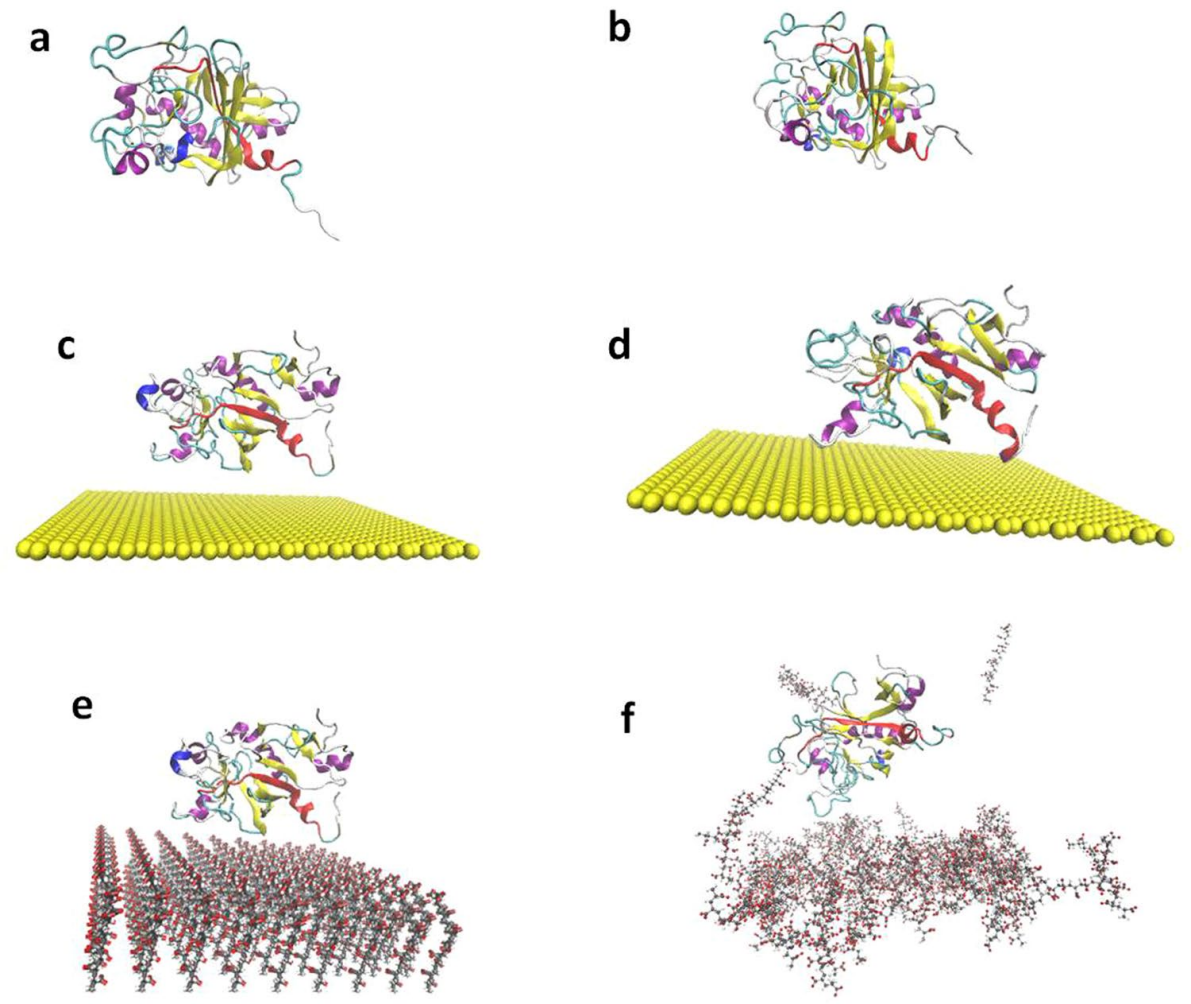
MD simulations to understand the nature of Fg interaction with gold NP surface. The structure of γFg protein (**a**) before and (**b**) after equilibration. The conformational changes of γFg on uncoated gold surface (**c**) before and (**d**) after equilibration (explicit water molecules are not shown here). The γFg protein structure on PAA coated gold surface (**e**) before and (**f**) after simulation, gold surfaces are not shown.

MD simulations were used to understand the nature of the interaction between Fg and gold NP’s (both PAA coated and bare gold surface, Au(111)). Initially, the isolated γ-chain of fibrinogen (γFg) in water medium was optimized (Fig. 1a,b); subsequently, γFg was simulated onto the coated (with PAA) and uncoated [Au(111)] surface of gold NP’s in a distance of 25 A° from the gold surface to deduce the effect of surface on the conformational changes of γFg (Fig. 1c,e). The conformation changes of the Fg after interaction with the coated and bare gold surfaces have been depicted in Fig. 1d,f. Also a 2D plot to examine how the change in secondary structure of Fg due to Au(111) causes decreasing alpha helix content, is shown in SI (Fig. S1).

**Figure 2:**
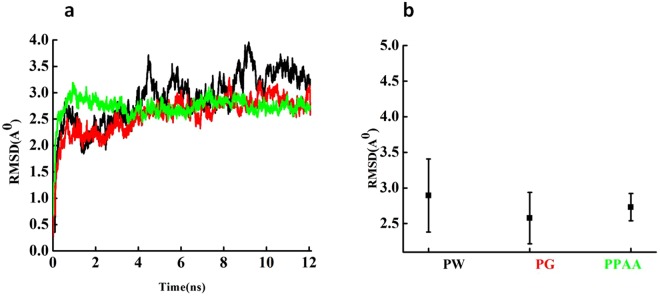
(**a**) RMSD of Fg (black), Fg over Au surface (red) and Fg over the PAA-coated Au surface (blue). (**b**) The average RMSD plus errors in each system, Fg protein with water (PW), Fg protein on the gold surface (PG) and Fg protein on the gold surface coated with PAA (PPAA)).

In MD simulation, the root-mean-square deviation (RMSD), radius of gyration (Rg) and solvent accessible surface area (SASA)^23^ were analyzed. To evaluate the equilibrium of the considered systems, RMSD of the Fg proteins was measured (Fig. 2a). After 12 ns of simulation, the RMSD remained constant between 2–3.5, 2–2.8 and 2–2.5 A° for Fg protein, conjugated Fg with bare and PAA coated gold surfaces, respectively. The small changes in RMSD indicated that Fg was undergoing a conformational change during the simulation (Fig. 2b). Indeed, both coated and bare gold surfaces demonstrated specific interactions with Fg chains, resulting in stable configuration on a given surface.

**Figure 3:**
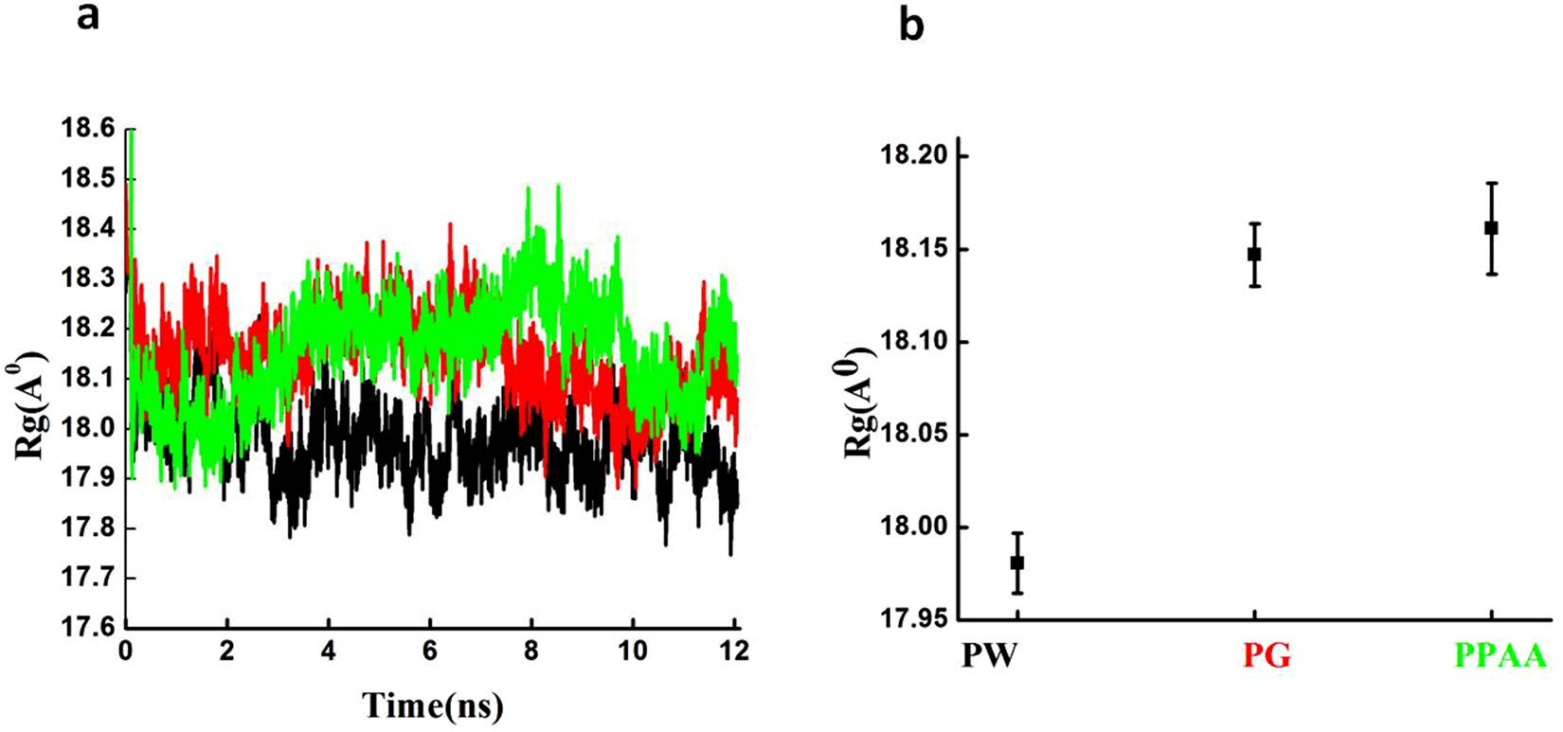
(**a**) Radius of gyration curves of Fg protein (black), Fg protein over Au surface (red) and Fg protein over the PAA-coated Au surface (blue). (**b**) The error bar in the average Rg in each system, Fg protein with water (PW), Fg protein on the gold surface (PG) and Fg protein on the gold surface coated with PAA (PPAA)).

To estimate the compactness of Fg on the gold surface, Rg was calculated (Fig. 3). Considering the Fg proteins size range, those with a higher Rg have less tight packing, while lower Rg suggests tighter packing. The Rg graphs clearly indicated that Fg structure got conjugated to both coated and bare gold surfaces and acquired an expanded flexible conformation. Figure 3b displays the error bar in average of Rg, indicating the validity of calculated Rg values.

**Figure 4:**
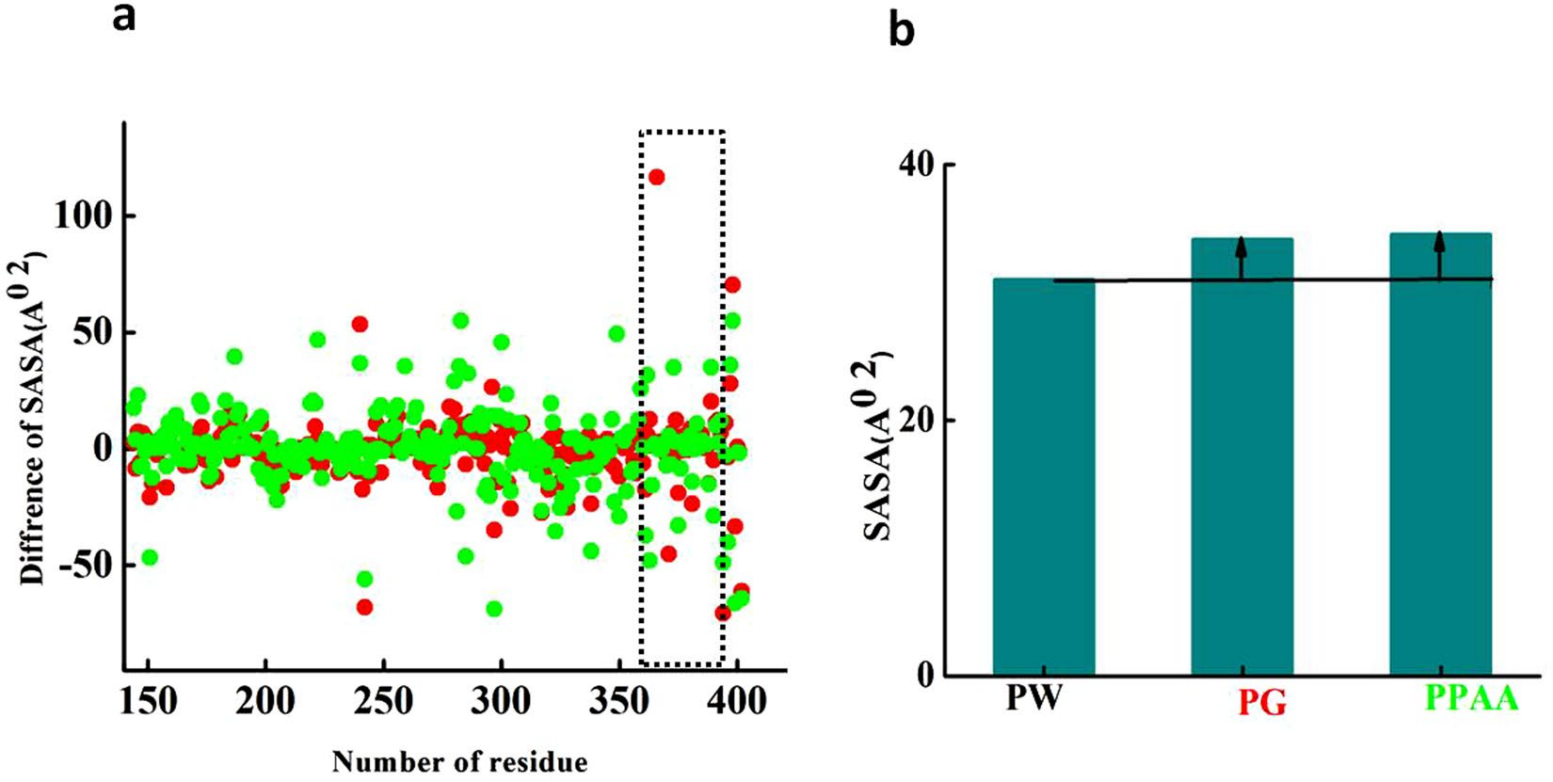
(**a**) Difference of SASA of γFg in solution and over the surfaces, Au (green), PAA- coated Au (red), (**b**) the average of SASA on each system of PW, PG and PPAA.

These observations were further validated by SASA analysis. Figure 4 shows the changes in difference of SASA between Fg and Fg over the Au(111) surface. The binding of Fg over gold surface caused the exposure of short sequences 377 -380 -383 -384 -385 -386 -387 -391 -392 -393 -395 (Fig. 4), that were also experimentally reported by Minchin *et al*. on PAA coated Au(111) NP’s^3^. These results demonstrated that the bare Au(111) surface could also induce the conformational change of Fg chain, which finally exposes these sequences. Interestingly, in the case of interaction between Fg and PAA-coated Au(111), the structural changes of PAA were also responsible for induction of the conformational changes of Fg and exposing its C-terminal sequences.

### Fibrinogen Binding to CTAB and Citrate Gold NPs

MD simulations clearly showed that, in addition to the PAA, the bare surface of gold can drive the conformational changes in Fg which is responsible for inflammatory responses. To confirm this even further, we hypothesized that ultra-small gold NPs (with the size of ~10 nm or smaller) with other surface coatings should also show the unfolding of Fg and release of inflammatory cytokines. This is mainly because of the disorganization of the surface coverage of gold NPs at this ultra-small size range^24–27^ which may increase the possibility of Fg interactions with the bare gold surface. Ligand dissolution when the gold NP’s are dispersed in culture may also open up binding sites for the Fg to interact with the gold surface. To test this hypothesis, we have synthesized both anionic citrate-coated (with size of ~5 nm) and cationic CTAB-coated gold NP’s (with size of ~10 nm) and probed their interactions with both Fg and smaller model peptides, using absorbance spectroscopy, ^1^H-NMR spectroscopy, circular dichroism measurements. Surface chemistry analysis on larger particles suggests that both capping agents cover the surface of gold NPs basically completely; however, the citrate coating has been shown to be variable in the citrate conformation and binding motifs, possibly existing as chelates within a multilayer^24–27^ With regards to CTAB, spherical particles are thought to be coated with a capping agent structure that is something between a CTAB monolayer and a CTAB bilayer^26^. Specifically, the CTAB layer is thought to be less well-organized on the surface of small spherical particles than it would be on the surface of a gold nanorod. Between the inherent organization of the capping agent on the metal core’s surface, and the possibility for capping agents to desorb from the particle surface under specific solution conditions^28^, Fg has several possible pathways to penetrate into the coating layer and interact with the metal surface. Previous studies have shown that citrate-coated gold NP’s are subject to aggregation in calcium-containing media (such as RPMI), which may be accompanied by ligand desorption. Absorbance spectroscopy analysis demonstrated that citrate gold NP’s underwent mild aggregation in media designed to simulate the ionic strength of RPMI (Fig. S5), which leaves open the possibility that citrate might desorb from the particle’s surface, opening up further opportunities for Fg to access the gold core. CTAB gold NP’s also show a change in their local dielectric environment and evidence mild aggregation when dispersed in minimal RPMI media (Fig. S6). Furthermore, small peptides (such as glutathione) seem to be capable of penetrating the CTAB layer, and adsorbing to the gold core, as indicated by both absorbance and 1H-NMR spectroscopy measurements (Figs S7 and S8).

**Figure 5:**
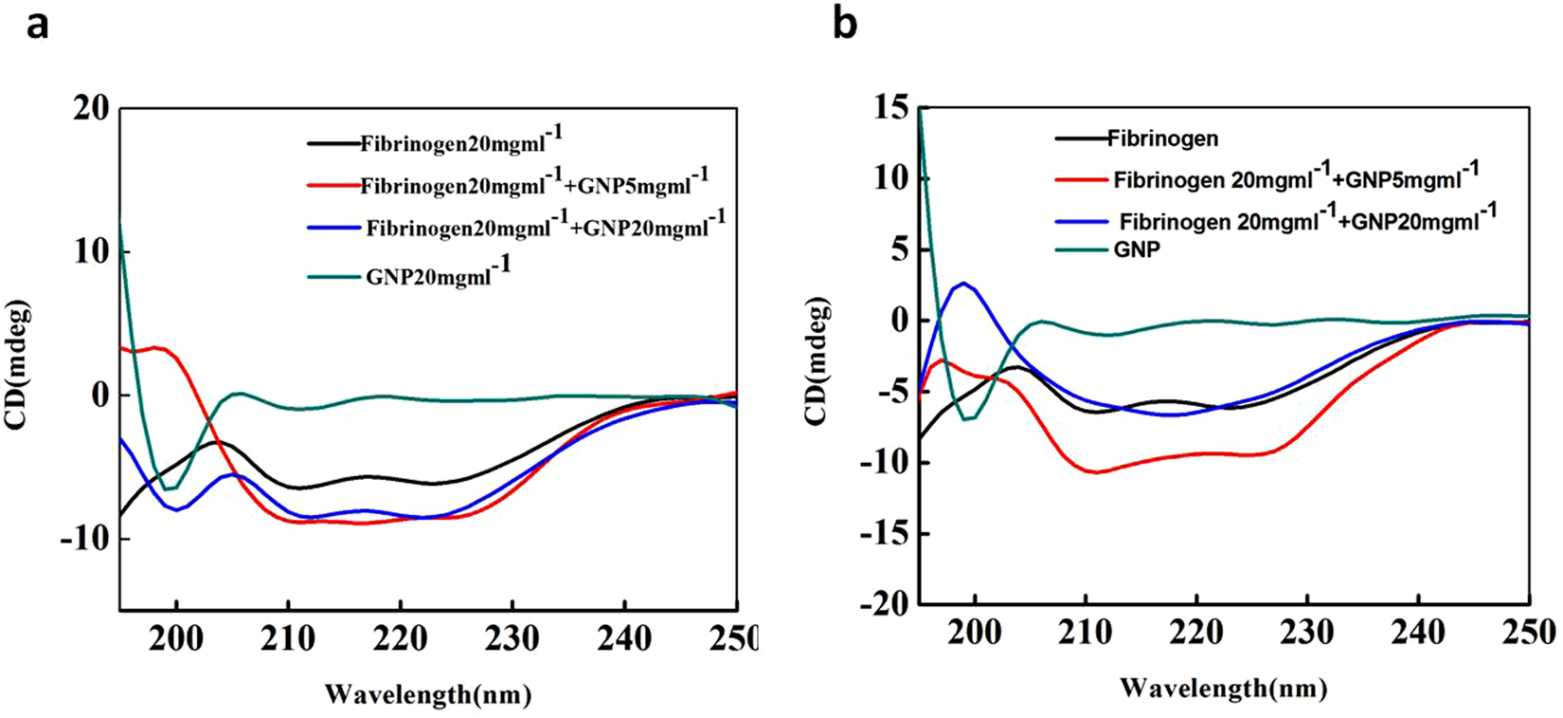
Circular dichroism (CD) spectra for Fg in the presence and absence of (**a**) anionic or (**b**) cationic gold NPs. By increasing the concentration of gold NPs, the ellipticity is decreased.

The prepared NP’s were fully characterized by transmission electron microscopy (TEM), dynamic light scattering (DLS), ζ-potential analysis, and UV/Vis spectroscopy and the corresponding results can be found in Fig. S2–4. UV-Vis and circular dichroism (CD)^29^ spectra were acquired after incubation of different concentrations of gold NPs with Fg. The intensity of the Surface Plasmon Resonance (SPR) band of gold NPs was enhanced with increasing the gold NP concentration (Fig. S9). The changes in secondary structure of the native Fg proteins and the Fg conjugated gold NPs were determined with CD spectroscopy, which showed that by increasing the gold NPs concentration, the changes in the secondary structure of Fg increased (ellipticity is decreased) (Fig. 5). This caused a reduction in ellipticity (less negativity) or decrease in the α-helix content for both anionic and cationic coatings. CD spectra of the interaction between Fg and several different concentrations of gold NP’s are shown in Fig. S10a,b.

### Cellular Uptake of Fibrinogen Conjugated Gold NPs

The surface charge of gold NP’s is a major factor dictating their cellular interactions^30^. For instance, Chol *et al*. studied the role of surface charge in internalization of gold NP’s^31^. They found that anionic gold NPs are absorbed to a much lower extent on the cell membrane than cationic NP’s^31^. To investigate whether surface charge was influencing the structure of Fg and increase the secretion of inflammatory cytokines, Mac-1-receptor-positive THP-1 cells and Mac-1-receptor-negative HL-60 cells, were incubated with Fg and the CTAB and citrate-coated gold NP’s. These cells were selected based on the recent findings on their suitability for monitoring integrin receptor activation^3^.

**Figure 6:**
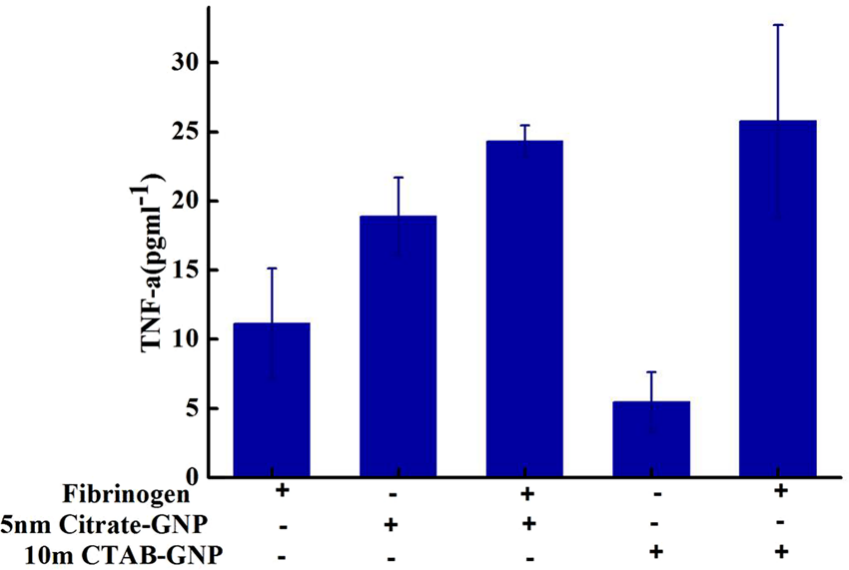
Treatment of HL-60 cell with complexes of Fg (20 µg mL^−1^) and gold NP (5 µg mL^−1^) induced the secretion of TNF-α. Cationic gold NPs induced TNF-α secretion to a higher level than anionic gold NPs, but even the latter effect was statistically significant. Asterisk indicate p < 0.05 compared to NP alone (respective control).

As shown in Fig. 6, in the presence of Fg, both the anionic and cationic gold NPs comparably induced the TNF-α paracrine factor. Citrate-coated anionic gold NP’s have lower ζ-potential and are expected to display low affinity binding to the cell, while CTAB-coated cationic gold NP’s have high ζ-potential and must exhibit higher affinity to the cell membrane. Since Fg has a negative charge at physiological pH (7.4), interaction of cationic gold NPs with Fg is believed to be mainly electrostatic. Indeed, the small changes in charge density can affect the interaction of Fg and gold NP’s. Presumably, in the interaction of anionic gold NP’s with Fg, the C-terminus of the protein which has a positive charge^32^, plays the role of binding site between the protein and the NP coating layer.

**Figure 7:**
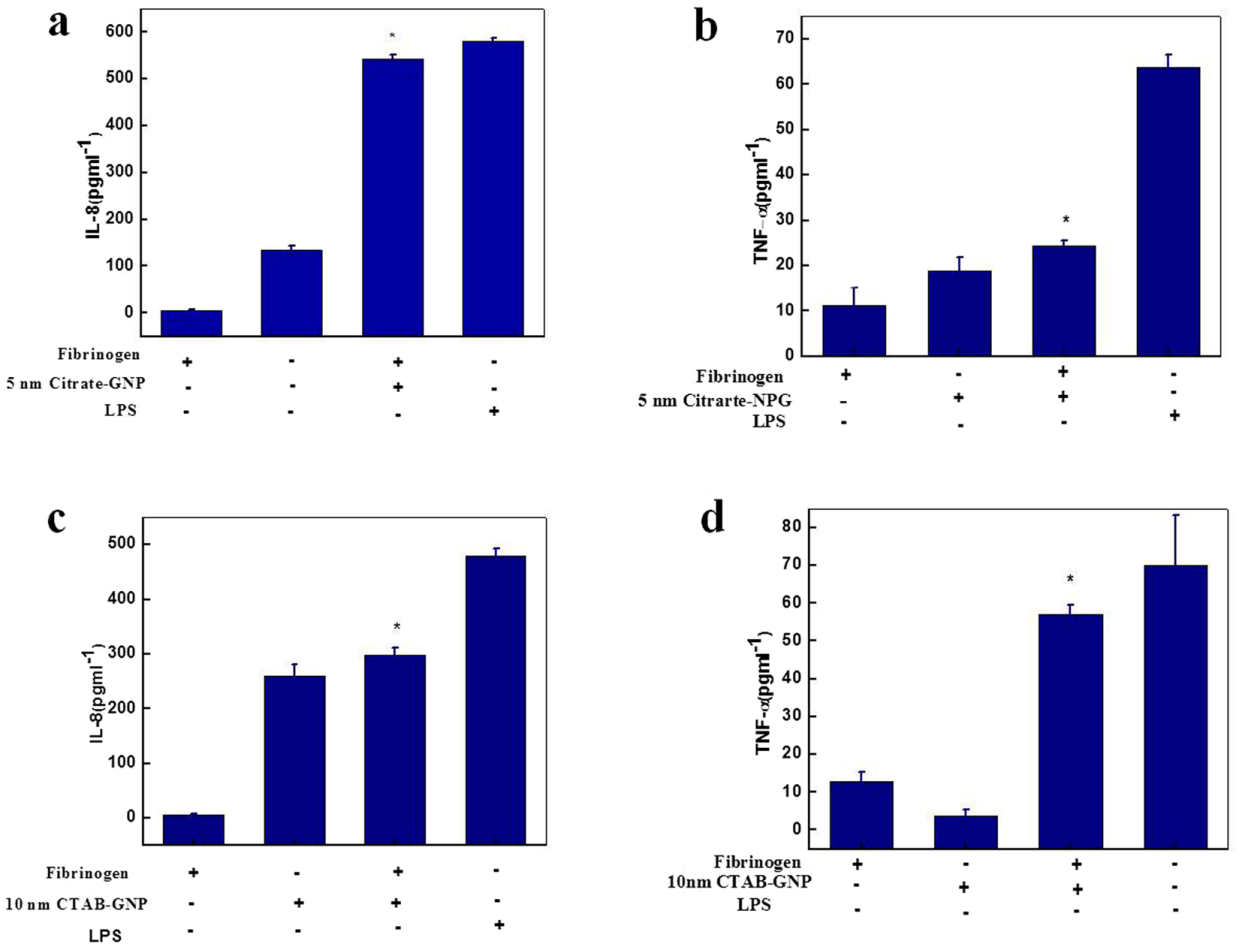
HL-60 cell with complexes of Fg (20 µg mL^−1^) and 5 nm citrate-gold NPs (5 µg mL^−1^) induced the secretion of (**a**) IL-8 and (**b**) TNF-α. THP-1 cell complexes of Fg (20 µg mL^−1^) and 10 nm CTAB-gold NP (20 µg mL^−1^) induced the secretion of (**c**) IL-8 and (**d**) TNF-α. Asterisk indicate p < 0.05 compared to NP alone (respective control). LPS as the positive control.

Figure 7 shows that in most cases, neither Fg nor NP alone altered cytokine release, but Fg-NP complexes increased IL-8 and TNF-α levels. Since the negative charge of anionic gold NP’s slightly decreases due to weak binding interactions between citrate and Fg^33^, gold surface mainly induces the unfolding of Fg, which promotes interaction with the receptor (Mac-1). Subsequently, the activation of Mac-1 leads to increased secretion of inflammatory cytokines (Fig. 7a,b). Due to the strong electrostatic interaction of CTAB gold NPs with fibrinogen^33^, gold surface and CTAB mutually contribute to Fg conformational changes and interaction with Mac-1 receptor, resulting in secretion of inflammatory cytokines (Fig. 7c,d).

As both NP types studied here induce conformational changes in Fg and can also promote inflammatory cytokine production in cells, it can be concluded that the effects of gold NPs on denaturation of Fg is coating type-independent, and mainly due to the interactions of the bare gold surface. These results suggest that gold NPs may cause unforeseen long-term *in vivo* inflammatory effects, as their surface coatings may be degraded *in vivo* and leave the bare surfaces accessible for inflammatory proteins such as Fg.

In summary, we demonstrated (using MD simulations) that the bare gold surface is the major player in interaction of gold NP’s with Fg, inducing conformational changes and finally driving the inflammatory response through Mac-1 receptor. The MD findings are further reinforced by the fact that for gold NP’s smaller than 10 nm, the coating is likely either partially removed from the surface of gold NP’s both *in vitro* and *in vivo*, or contains gaps due to the ligand’s binding geometry. This potentially leaves the NP surface accessible to Fg. Follow-up empirical experiments also confirmed that both cationic and anionic NP’s denature Fg, regardless of the type of coating used for coating or the surface charge. Similar mechanistic studies can be useful for studying the interactions of other NP’s with plasma proteins for assessing safety in nanotoxicology. These findings potentiate concerns about the unforeseen long-term *in vivo* inflammatory effects of gold NP’s.

## Methods

### Molecular Dynamic Simulations

MD simulation of Au(111) and γFg were performed with LAMMPS program, run efficiently on the computational cluster. The integration of the equations was done using the velocity Verlet algorithm with a time step of 1 fs. For preparation of the system, the crystal structure of a 30 kDa C-terminus γ-chain fragment of Fg was obtained from the Protein Data Bank (PDB ID:1FID). Fg has a net charge of −3 e.

MD simulations were carried out in two steps: a minimization of energy was employed to find the optimal orientations for protein on the surface for 2 ns in the NPT ensembles and atomistic MD simulation run for 10 ns in the NVT ensembles at T = 310 K. TIP3P water model^34^ as implemented in LAMMPS has been used in this process. During the simulation process, a Nose-Hoover thermostat^35^ was utilized to control the desired temperature. We positioned protein on a surface plane to increase the maximum number of possible contacts between the proteins and the Au surface. Periodic boundary condition in three directions was applied. Cut off of van der waals interaction was set to 8 A°, and 12 A° for electrostatic interactions. The gold atoms were kept fixed to accelerate computation. We used CHARMM27^10^ force field for proteins because this force field is efficient due to its parameterization. As the interaction with metal surface is not provided by the CHARMM27, terms of non-bonded interaction of the solvent and protein with the gold atoms was added. The potential between atoms in the molecule and metal surface were represented by a Lennard-Jonnes 6–12 potential^36^.

1 $${\rm{U}}=4{\rm{\varepsilon }}[{({\rm{\sigma }}/{\rm{r}})}^{12}-{({\rm{\sigma }}/{\rm{r}})}^{6}]$$

where ε is the depth of the potential, σ is the finite distance where the inter particle potential is zero, r is the distance between atoms. ε and σ values were taken from GOLP-CHARMM^20^ force field, where r = 3.8005 A^0^ and ε = 0.1147 kcal mol^−1^. Electrostatic interactions were treated using particle mesh Ewald method^37^.

### Experimental section

#### Materials

Hydrogen tetrachloroaurate (HAuCl_4_•3H_2_O), cetyltrimethylammonium bromide (CTAB), L-ascorbic acid, glutathione, and Fg (from human plasma) were purchased from Sigma. Sodium borohydride (NaBH_4_) and trisodium citrate were bought from Merck. Throughout the NPs preparation process, ultrapure deionized water (DI; Continental Water Systems) was used.

#### Citrate-coated gold NPs

Citrate-coated gold NP’s were synthesized according to the method described previously^38,39^. In brief, 0.05 mmol of trisodium citrate was added to solution containing 240 mL of HAuCl_4_ solution (0.21 mmol L^−1^) and 5 mL of an ice-cold NaBH_4_ solution (0.1 mol L^-1^). Afterwards, the resulting solution was stirred at room temperature overnight. The gold NPs were washed three times with DI water by sequential centrifugation at 45000 g for 20 min, 250000 g for 10 min and 20000 g for 5 min.

#### CTAB-coated gold NP’s

CTAB-coated gold NP’s were prepared according to approach reported by Murphy *et al*.^39^. The seed solution was prepared by suspending 0.6 mL of freshly prepared NaBH_4_ solution (0.01 mol L^−1^) into 20 mL aqueous solution contains HAuCl_4_ (0.25 mmol L^−1^) and trisodium citrate (0.25 mmol L^−1^). To prepare the CTAB-capped gold NP’s, 0.050 mL^−1^ of ascorbic acid (0.1 mol L^−1^) was suspended in 7.5 mL of growth solution (200 mL 0 f 0.08 mol L^−1^ CTAB and 0.25 mmol L^−1^ HAuCl_4_). Afterwards, 2.5 mL of seed solution was added and stirred continuously for 10 min. It is noteworthy to mention that before using the NPs, they were washed three times with DI water by sequential centrifugation at 50000 g for 20 min, 30000 g for 10 min and 20000 g for 5 min.

#### Protein corona

Fg was dissolved in PBS at different concentrations. The gold NP’s (20 g mL^−1^ and 5, 20, 25 g mL^−1^) were incubated with Fg for 60 min at 37 °C and then centrifuged at 1000 g, for 15 min in room temperature to collect the corona-coated gold NP’s. The corona-coated NPs were re-suspended in PBS and centrifuged under the same conditions. The process was repeated three times to remove the loosely attached proteins on NP’s

#### UV/Vis spectra

The UV-visible spectra of corona-coated gold NP’s and Fg were measured at 200–700 nm wavelength range with a Shimadzu UVmini-1240 UV-Vis Spectrophotometer.

#### ^1^H-NMR Spectroscopy

^1^H-NMR spectra were obtained using a JEOL Delta ^1^H-NMR spectrometer (300 MHz). 1H-NMR spectra of CTAB gold NPs, CTAB, and glutathione spectra were taken in D_2_O.

#### Circular dichroism (CD)

The changes in secondary structure of the corona Fg conjugated gold NP’s solution were determined by CD spectroscopy at the 190–260 nm wavelength range with JASCO J-715 spectropolarimeter. The JASCO program was used for analysis of spectra.

#### Cell culture experiments

HL-60 and THP-1 were obtained from Pasteur Institute of Iran (IPI) and were cultured in RPMI1640 culture medium, 10% fetal bovine serum and penicillin/streptomycin at 37 °C in 5% CO_2_. The cells were treated with different concentrations of Fg-coated gold NP’s (20 µg mL^−1^and 25, 20, 5 µg mL^−1^ respectively) in serum-free medium. The cells were incubated with Fg or NP’s separately for control experiments. After 6 h incubation, supernatants were removed and cytokine levels were determined using an Elisa kit (eBioscience). Information of HL-60 cells: [Cell Shape: Ovoid or Round; Cell Type/Origin/Size: Monocyte/Human/ 9 to 25 μm; Cell Passage: 6th; Cell Sex: Female; Cell Transition: Myeloblastic or Promyelocytic] and THP-1 cells: [Cell Shape: Round; Cell Type/Origin/Size: Monocyte/Human/ 18-23 µm; Cell Passage: 7th; Cell Sex: Male; Cell Transition: Monocytic].

## Electronic supplementary material

Supplementary Information
