# Supplementary material for: Bare surface of gold nanoparticle induces inflammation through unfolding of plasma fibrinogen
Source: Sci Rep. 2018 Aug 22;8:12557. doi: 10.1038/s41598-018-30915-7 (PMC6105630; doi:10.1038/s41598-018-30915-7)
Supplement: Supplementary file 1 — Supplementary Information [file 41598_2018_30915_MOESM1_ESM.pdf]

Supplementary Information

Bare surface of gold nanoparticle induces inflammation through unfolding of plasma fibrinogen

Bahar Kharazian[1], Samuel E. Lohse[2], Forough Ghasemi[3], Mohamad Raoufi[4,5], Amir Ata Saei[6], Fatemeh Hashemi[4], Fakhrossadat Farvadi[4], Reza Alimohamadi[7], Seyed Amir Jalali[8], Mohammad A. Shokrgozar[9], Nasser L. Hadipour[1], Mohammad Reza Ejtehadi[10*], Morteza Mahmoudi[11*]*

[1]Department of Chemistry, Tarbiat Modares University, P. O. Box 14115-175, Tehran, Iran

[2]Physical and Environment Sciences Program, Colorado Mesa University, Grand Junction, Colorado 81501, United States

[3]Department of Chemistry, Sharif University of Technology, Tehran, 11155-9516, Iran

[4]Department of Nanotechnology and Nanotechnology Research Center, Faculty of Pharmacy, Tehran University of Medical Sciences, Tehran, Iran

[5]Department of New Materials and Biosystems, Max Planck Institute for Intelligent Systems, Heisenbergstraße 3, D-70569 Stuttgart, Germany

[6]Division of Physiological Chemistry I, Department of Medical Biochemistry and Biophysics, Karolinska Institutet, Scheelesväg 2, SE-17 177 Stockholm, Sweden

[7]Department of Immunology, School of Medicine, Shahid Beheshti University of Medical Sciences, Tehran, Iran

[8]Departman of Immunology, Shahid Beheshti University of Medical Sciences, Tehran, Iran

[9]National Cell Bank, Pasteur Institute of Iran, Tehran, Iran

[10]*Department of Physics, Sharif University of Technology, P. O. Box 11155-9161, Tehran, Iran and Center of Excellence in Complex Systems and Condensed Matter (CSCM), Sharif University of Technology, Tehran 1458889694, Iran*

[11]*Center for Nanomedicine and Department of Anesthesiology, Brigham and Women's Hospital, Harvard Medical School, Boston, Massachusetts 02115, United States*

[*]*Corresponding Author: (NLH) E-mail: hadipour@modares.ac.ir; (MRE) E-mail: ejtehadi@sharif.edu; (MM) E-mail: mmahmoudi@bwh.harvard.edu*

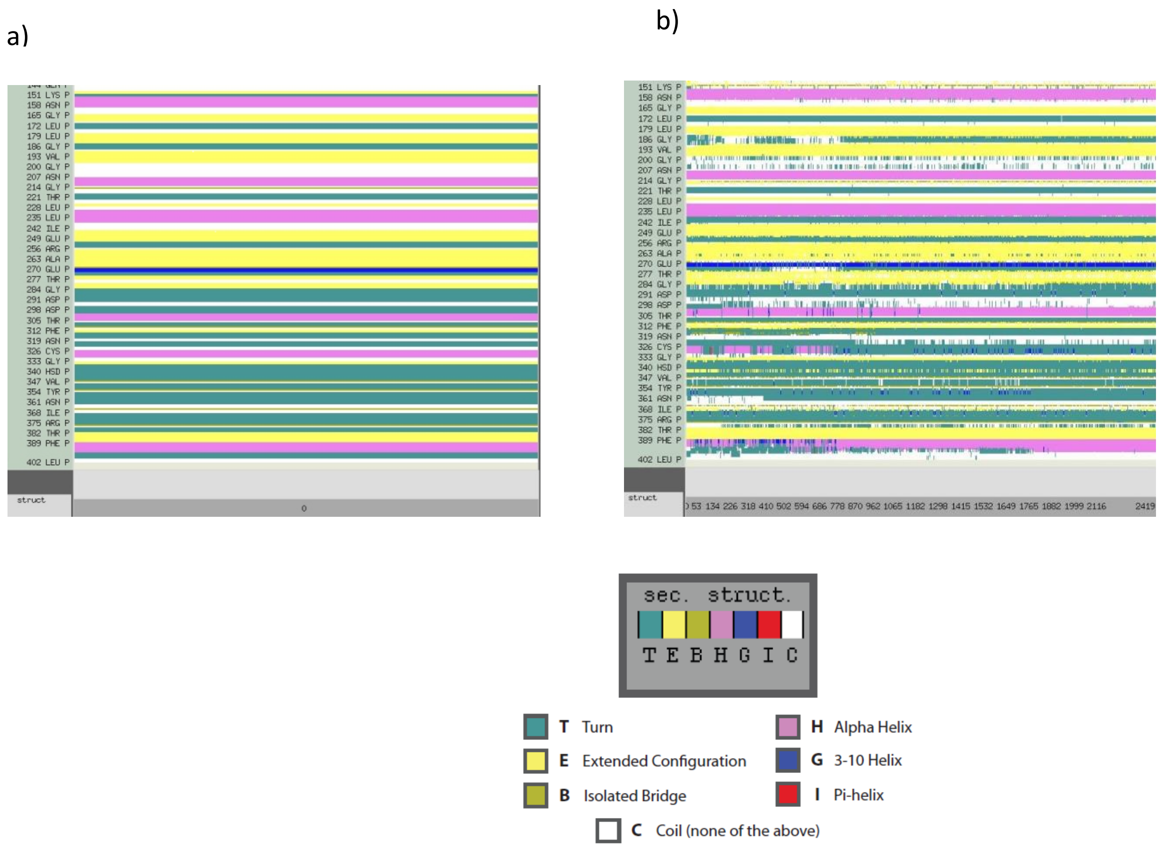

Figure S1: Secondary structure for all residues of Fg for all frames. a) Shows the system before the local structure changes b) Shows the system afterwards. Color key for secondary structure plots.

Methods:

Characterization of NPs:

The purified gold NP's were characterized by transmission electron microscopy (TEM), dynamic light scattering (DLS), ζ-potential analysis, and UV/Vis spectroscopy. TEM images (Figure S2) revealed that the synthesized NP's have the desired size. The citrate gold NP's were found to have a mean diameter of 5 ± 2 nm (n = 483). The CTAB gold NP's were more polydisperse, and determined to have a mean diameter of 10 ± 5 nm (n = 201). DLS measurements of the

hydrodynamic diameters (D_H) of gold NP's are presented in Figure S3. DLS analysis for both particle types (in deionized water) shows that the D_H of NPs is ~ 8 nm, which is consistent with the gold NP core size, as determined by TEM analysis. The UV-Vis spectra of the gold NP's show that gold NP's exhibit a characteristic plasmon absorbance peak in the visible range (522 and 505 nm for CTAB- and citrate-coated gold NP's, respectively; Figure S4). ζ-potential analysis indicated that (in deionized water) citrate-coated gold NP's possessed a negative surface charge of -21.6 mV, while CTAB-coated gold NP's had a positive surface charge of 27.0 mV, as expected.

Supplementary Figures:

a) b)

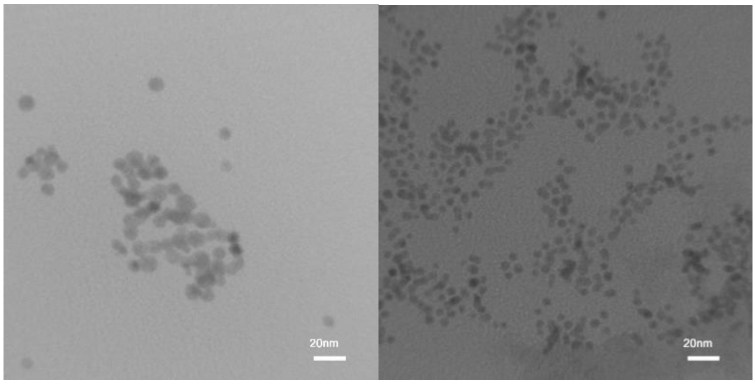

Figure S2: TEM image of AuNP’s capped with a) CTAB b) citrate

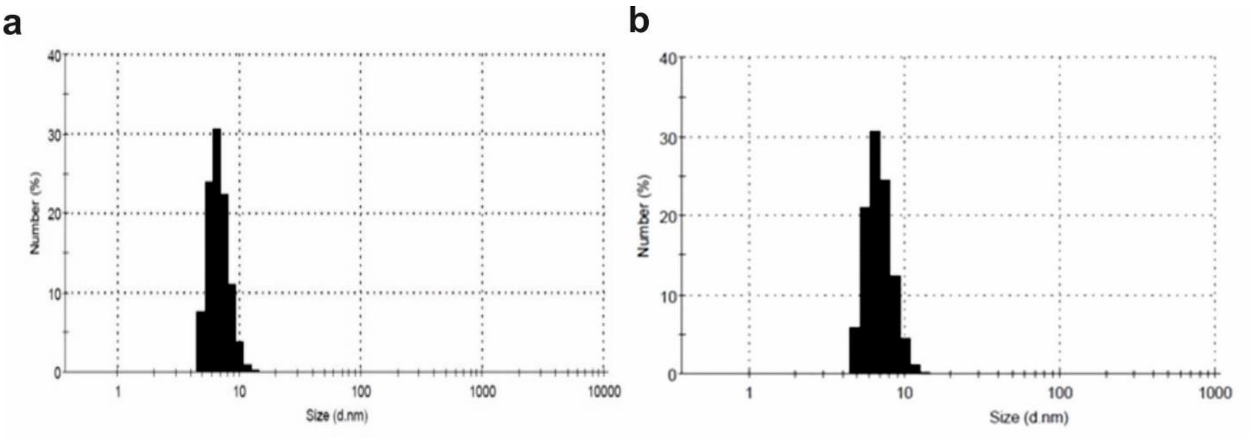

Figure S3: Particle size distribution measured by DLS of a) CTAB- and b) citrate-coated AuNP’s

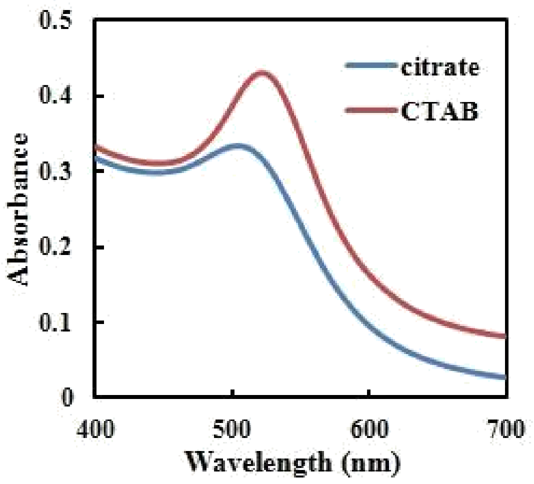

Figure S4: UV-Vis spectra of AuNP's

Stability of Gold NPs in Reduced RPMI Media. In order to explore the stability of both the CTAB- and citrate-goldNPs in RPMI medium, including the possibility for peptides to interact with the goldNP surface, citrate and CTAB gold NP's ([gold NP] = 1.0 nM) were dispersed in a medium that contained the typical cations and anions that are found in the RPMI media, with none of the proteins or biomolecules that are typically included in the medium (minimal RPMI medium). The ionic compounds used to simulate the RPMI media were: $Ca(NO_3)_2 \bullet 4H_2O$ (0.1 g/L), $MgSO_4$ (0.0488 g/L), KCl (0.4 g/L), NaCl (6 g/L), K_2HPO_4 (0.8 g/L), and $NaHCO_3$ (2 g/L). The stability of both gold NPs in RPMI was then monitored using absorbance spectroscopy. The absorbance data for the citrate gold NP's (Figures S5) shows that the surface plasmon absorbance of the gold NP's quickly broadens, and the baseline raises over the early analysis times. This suggests that the Cit gold NP's undergo some mild aggregation when placed in the minimal RPMI medium, which is likely primarily due to charge screening by ions in the RPMI medium. However, this aggregation is likely also either partially due to (or accompanied by) the desorption of citrate from some portion

of the particle surface. Any possible ligand desorption could open up further sites for fibrinogen to access the gold core of the particle.

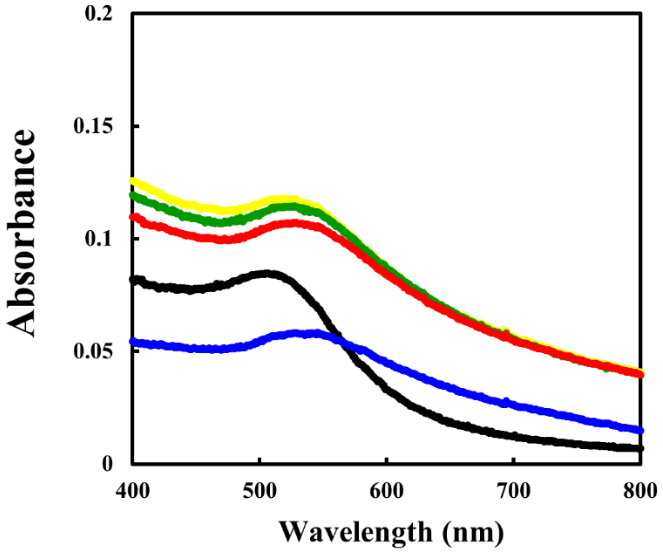

Figure S5. Absorbance spectra of Cit gold NPs dissolved in a minimal RPMI medium over time. 0 min (black trace). 5 min (red trace). 10 min (green trace). 15 min (yellow trace). 60 min (blue trace).

Absorbance spectroscopy analysis of the CTAB gold NP's also suggests that while the CTAB-AuNPs show good stability in the minimal RPMI media, the CTAB capping agent layer affords sufficient access for at least small peptides (such as glutathione) to interact closely with the gold NP's surface. Absorbance spectroscopy data for the CTAB AuNP's ([gold NP] = 1.0 nM) shows that the SPR λ_{max} is only mildly red-shifted when the gold NPs are dispersed in minimal RPMI, consistent with a change in the particle's dielectric environment, and (possibly) with mild desorption of CTAB from the gold NP core's surface (Figure S6).[1] The λ_{max} undergoes a shift in

position from 528 nm to 531 nm in this case. If a low molecular weight peptide, such as glutathione (GSH) is added to this solution, the λ_{max} shifts slightly further to 533 nm, consistent with a further change in the particle's dielectric environment, and potentially consistent with the glutathione penetrating the CTAB layer and binding to the gold NP surface.

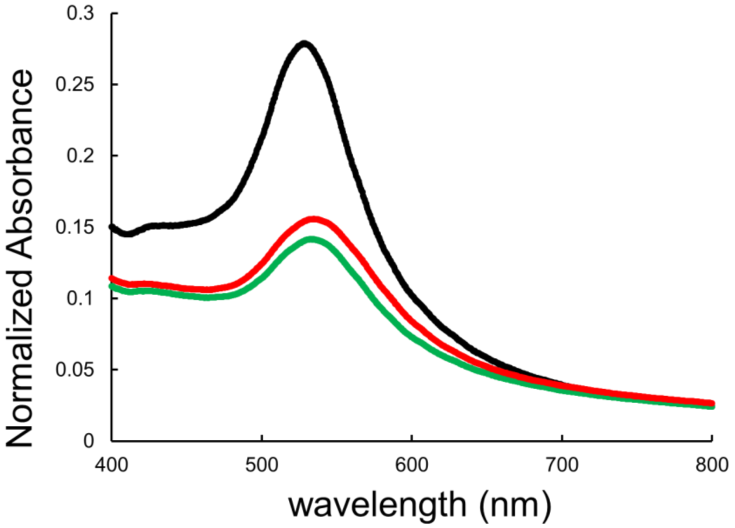

Figure S6. Absorbance spectra of CTAB AuNPs dispersed in minimal RMPI medium. (Black trace) CTAB AuNPs in deionized water, (Green trace) CTAB AuNPs in RPMI, (Red trace) CTAB AuNPs in RPMI and 1 mM glutathione. Spectral data were normalized at λ = 800 nm.

Small thiol-containing peptides, such as glutathione, have previously been shown to displace ligands either chemically bound or physically adsorbed to gold nanoparticles.[2,3] It seems likely that larger thiol-containing proteins (such fibrinogen) could also participate in these types of ligand-exchange reactions, as well, although electrostatic interactions between the ligand and protein may restrict protein access to the gold NP surface.[4] While absorbance spectroscopy measurements strongly indicate that glutathione interacts with the CTAB gold NP's in RPMI medium, the absorbance spectroscopy data alone does not necessarily demonstrate that the peptide penetrates the adsorbed CTAB and binds to the gold core in this case. Therefore, we also

investigated the binding of glutathione to the gold NP surface using ^{1}H-NMR spectroscopy (Figures S7, S8).

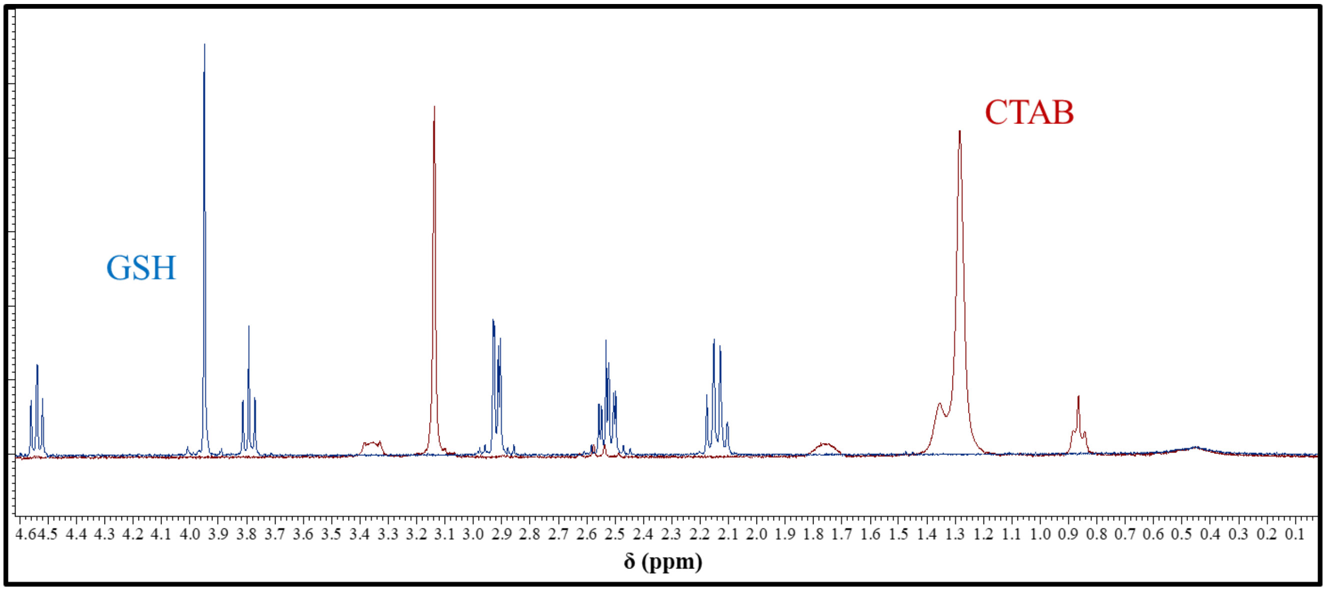

Figure S7. ^{1}H-NMR spectra (300 MHz) of aqueous CTAB (10.0 mM, red trace) and GSH (1.0 mM, blue trace) solutions.

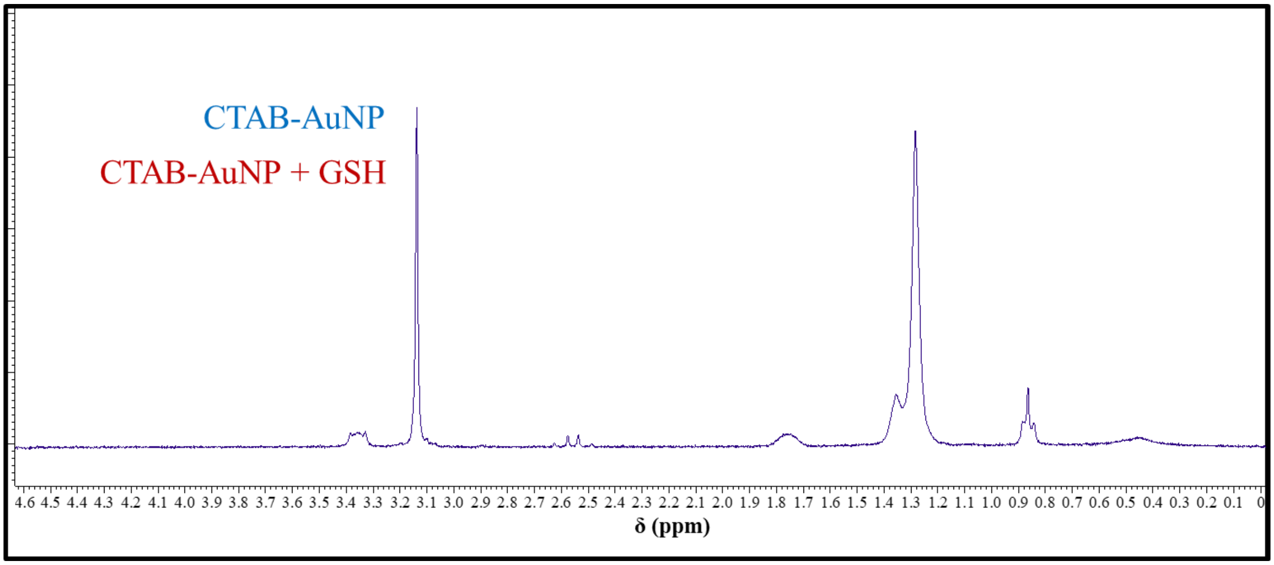

Figure S8. ^{1}H-NMR spectra (300 MHz) of CTAB-goldNPs dispersed in minimal RPMI media (blue trace), and CTAB-goldNPs in the presence of 1.0 mM GSH (red trace). pH = 6.7, [gold NPs] ~ 30.0 nM.

^{1}H-NMR analysis of the CTAB gold NPs dispersed in minimal RPMI (D_2O) shows weak, but discernable signals for the CTAB protons, which is consistent with organic molecules adsorbed to the surface of gold NP's, and only minimal amounts of free ligand in solution.[5] When GSH is added to the gold NP solution ([GSH] = 1.0 mM) in RPMI, the glutathione peaks are not apparent in the spectrum, despite the fact that GSH peaks are clearly present in the free ligand spectrum at the same concentration (Figure S7). This suggests that the glutathione may indeed penetrate the adsorbed CTAB layer, and bind to the gold NP surface, which would broaden the ^{1}H-NMR peaks associated with GSH, minimizing their appearance in the NMR spectrum.

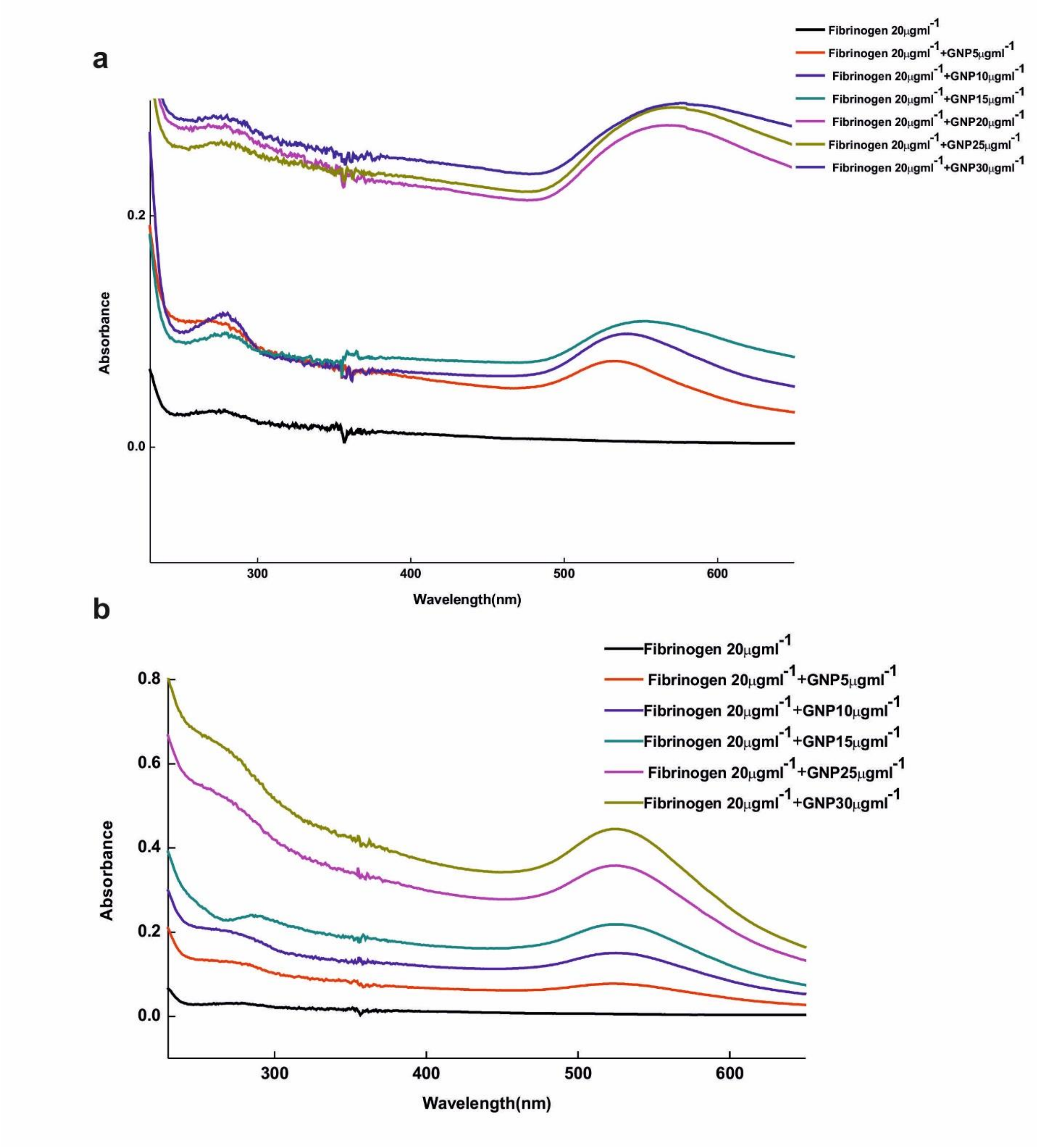

Figure S9: UV-Vis spectra of Fg conjugate with a) CTAB- and b) citrate-coated AuNP's in different concentrations

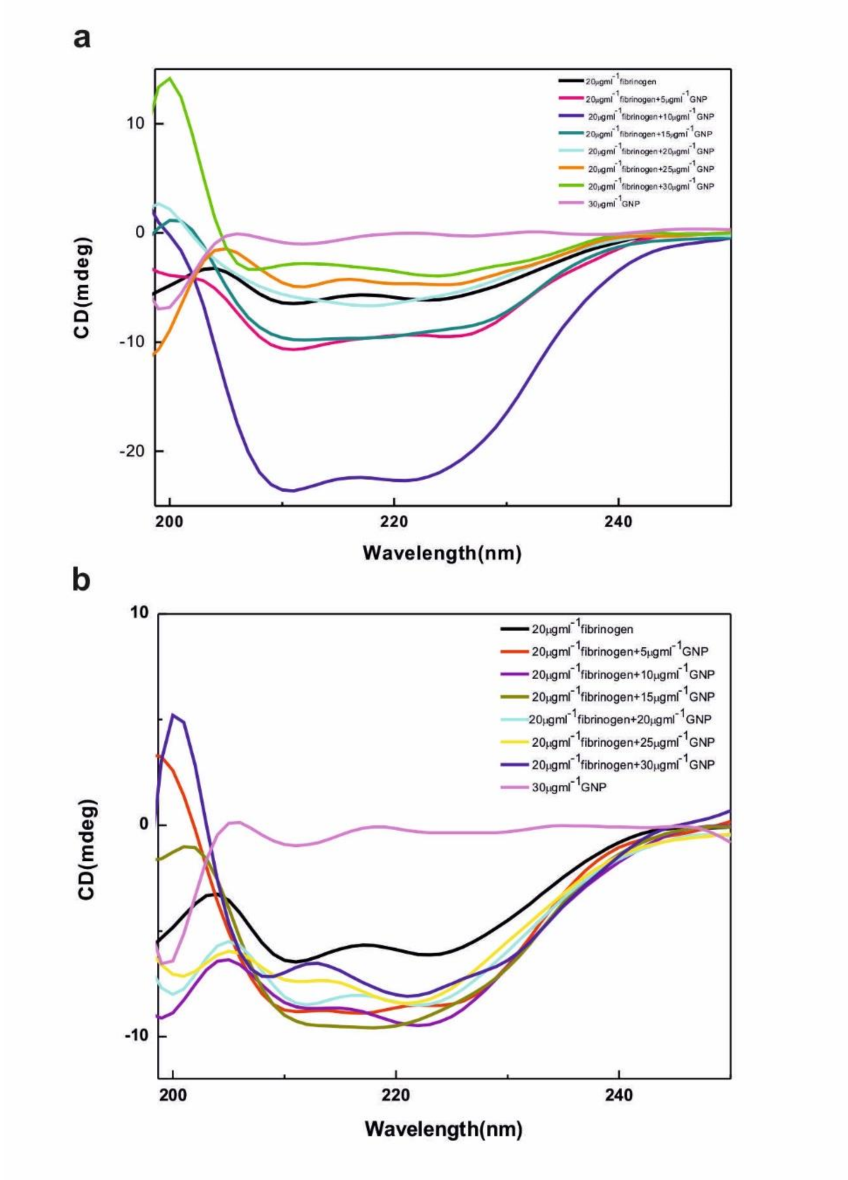

Figure S10: Circular dichroism (CD) spectra for Fg in the absence and presence of a) CTAB- and b) citrate-coated AuNP’s in different concentrations

References

1. Xu, J.X.; Siriwandara, K.; Zhou, Y.; Zou, S.; Zhang, D. Quantification of gold nanoparticle ultraviolet-visible extinction, absorption, and scattering cross section spectra and scattering depolarization spectra: The effects of nanoparticle geometry, solvent composition, ligand functionalization, and nanoparticle aggregation. *Anal. Chem*. **2017**, *Advance article online*.
2. Hong, R.; Han, G.; Fernandez, J.M.; Kim, B,J; Forbes, N.S.; Rotello, V.M. Glutathione-mediated delivery and release using monolayer protected nanoparticle carriers. *JACS* **2006**, *128*, 1078-1079.
3. Larson, T.A.; Joshi, P.P.; Sokolov, K. Preventing protein adsorption and macrophage uptake of gold nanoparticles via a hydrophobic shield. *ACS Nano* **2012**, *6*, 9182-9190.
4. Brewer, S.H.; Glomm, W.R.; Johnson, M.C.; Knag, M.K.; Franzen, S. Probing BSA binding to citrate-coated gold nanoparticles and surfaces. *Langmuir* **2005**, *21*, 9303-9307.
5. Sweeney, S.F.; Woehrle, G.H.; Hutchison, J.E. Rapid purification and size separation of gold nanoparticles via diafiltration. *J. Am. Chem. Soc.* **2006**, *128*, 3190-3197.
